# Selection of Higher Order Regression Models in the Analysis of Multi-Factorial Transcription Data

**DOI:** 10.1371/journal.pone.0091840

**Published:** 2014-03-21

**Authors:** Olivia Prazeres da Costa, Arthur Hoffman, Johannes W. Rey, Ulrich Mansmann, Thorsten Buch, Achim Tresch

**Affiliations:** 1 Institute for Medical Microbiology, Immunology and Hygiene, Technische Universität München, Munich, Germany; 2 St. Mary's hospital, Department of Medicine, Frankfurt, Germany; 3 Medical Department, Johannes Gutenberg University, Mainz, Germany; 4 Institute for Medical Informatics, Biometry and Epidemiology (IBE), Ludwig-Maximilians-Universität München, Munich, Germany; 5 Department of Plant Breeding and Genetics, Max Planck Institute for Plant Breeding Research, Cologne, Germany; 6 Institute for Genetics, University of Cologne, Cologne, Germany; Philipps University, Germany

## Abstract

**Introduction:**

Many studies examine gene expression data that has been obtained under the influence of multiple factors, such as genetic background, environmental conditions, or exposure to diseases. The interplay of multiple factors may lead to effect modification and confounding. Higher order linear regression models can account for these effects. We present a new methodology for linear model selection and apply it to microarray data of bone marrow-derived macrophages. This experiment investigates the influence of three variable factors: the genetic background of the mice from which the macrophages were obtained, *Yersinia enterocolitica* infection (two strains, and a mock control), and treatment/non-treatment with interferon-γ.

**Results:**

We set up four different linear regression models in a hierarchical order. We introduce the eruption plot as a new practical tool for model selection complementary to global testing. It visually compares the size and significance of effect estimates between two nested models. Using this methodology we were able to select the most appropriate model by keeping only relevant factors showing additional explanatory power. Application to experimental data allowed us to qualify the interaction of factors as either neutral (no interaction), alleviating (co-occurring effects are weaker than expected from the single effects), or aggravating (stronger than expected). We find a biologically meaningful gene cluster of putative C2TA target genes that appear to be co-regulated with MHC class II genes.

**Conclusions:**

We introduced the eruption plot as a tool for visual model comparison to identify relevant higher order interactions in the analysis of expression data obtained under the influence of multiple factors. We conclude that model selection in higher order linear regression models should generally be performed for the analysis of multi-factorial microarray data.

## Introduction

Gene expression is the result of a multitude of different mechanisms whose effects do not simply add up, but show complex interactions. The analysis of the biological processes underlying gene expression requires appropriate methodological approaches. This paper presents a simple tool to tackle these challenges using as an example the transcriptional response of the genetic background of mice upon interferon-gamma (IFN-γ) stimulation.

**Figure 1:**
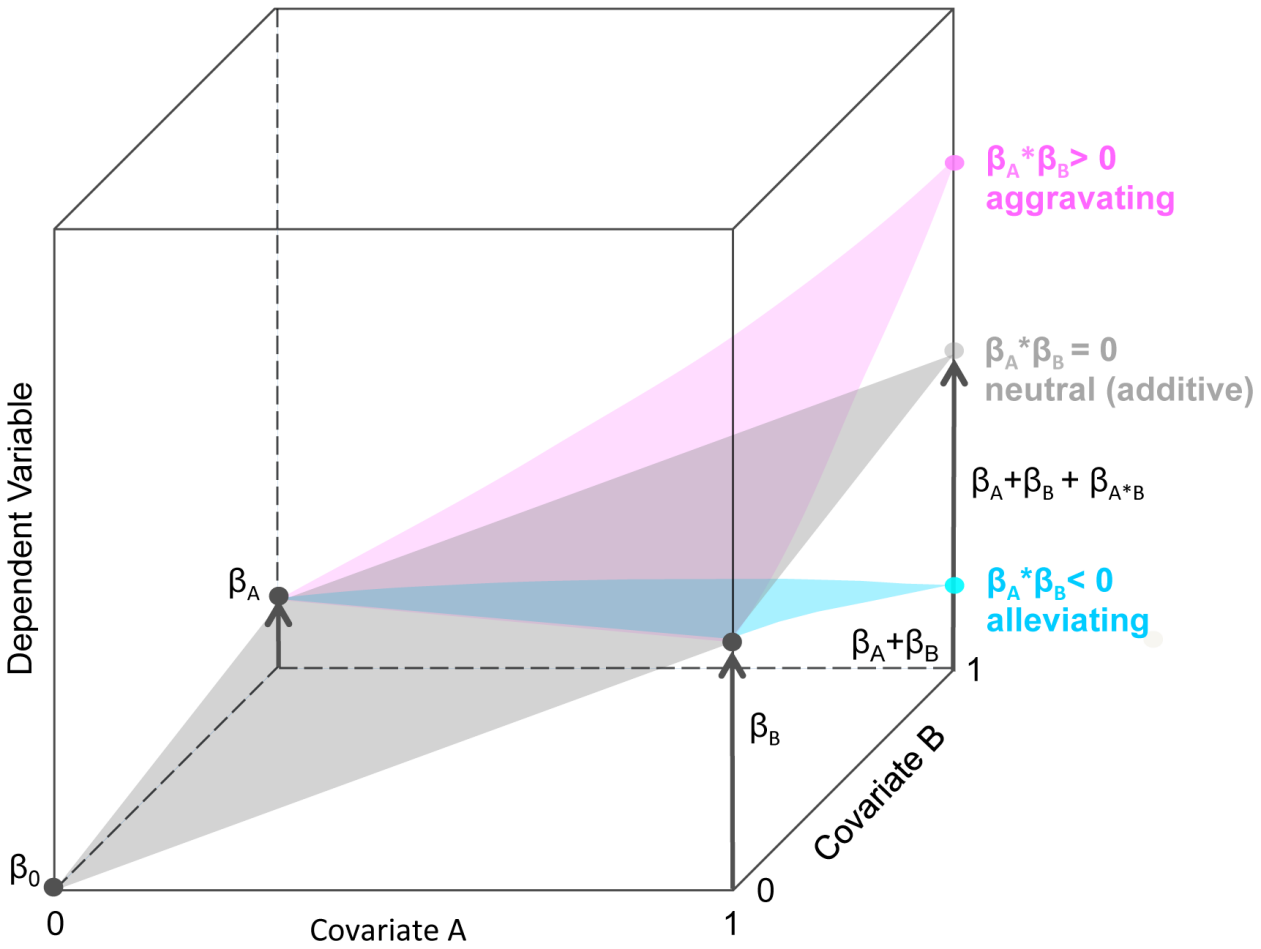
Interaction effects calculated by multiple linear regression. This schematic visualization of second order linear regression models interaction effects. The diagram of the linear regression model includes two main covariates (strain *H* and stimulation with *Γ*) and their interaction covariate *H∶Γ*. The main covariates can assume two values (*H*: C57BL/6 or BALB/c; *Γ*: IFN-γ stimulation or no stimulation). The arrows indicate the estimated effects β. The pink and turquoise arrows reflect the aggravating or alleviating interaction effects as deviations from the additive model. A second order linear model can dissect the effects arising from two perturbations and their interaction by looking at the magnitude and significance of its regression covariates. Most importantly, the interaction covariate can indicate either an alleviating (weaker than expected from the single intervention effects) or aggravating (stronger than expected) interaction. The linear model includes two main covariates *H* and *Γ* and their interaction covariate *Η∶Γ*.

Traditionally, the analysis of transcriptional regulation has been performed on the level of individual TF-target pairs. The advent of genome-wide transcription measurements provided a comprehensive look at signaling processes. The most widely used standard for the analysis of transcription data is linear regression as implemented, e.g., in the Limma package [1]. Linear regression quantifies gene by gene the individual effect that certain factors, so-called covariates, have on gene expression. Examples for covariates are gene deletion, environmental stress, or cytokine stimulation. Usually, it is assumed that the covariates contribute independently, e.g., additively, to the expression outcome. This leads to a so-called first order linear regression model, in which one effect (main effect) is calculated for each covariate. While this type of analysis has been extremely successful, it often constitutes an unjustified simplification and the assumption of additivity is often violated. The most extreme examples of such violations are so-called synthetic lethal interactions, where gene deficiency of one or the other gene has no or mild effects, but the double gene deficiency is lethal [2], [3]. Non-additivity can also occur at the level of gene expression. There, higher order interaction and effect modification typically arise from cooperation or competition of transcription factors at their target genes [4]. But how can we reliably identify such a complex interplay between covariates for many genes at a time? Classical methods such as adjusted R^2^, Akaike information criterion (AIC) and more complex strategies like global tests such as GlobalAncova [5] or Goeman's global test [6] estimate the effect of a covariate over all genes simultaneously and give a global and abstract assessment on which factors determine the observed expression profiles. Linear models can be enhanced by the incorporation of interaction terms, whose magnitude and significance tell us if and how gene expression deviates from additivity of the main effects as assumed by the first order linear model. A non-zero interaction effect indicates that a simple additive model is inappropriate. Interactions can be classified into one of the following groups (Figure 1) [7]: an interaction effect between two covariates is called alleviating (aggravating, neutral), if the effect of the joint action of the covariates is weaker than (stronger than, identical to) the sum of the individual effects of these covariates. Interaction models have been used to study the effect of combined gene-deficiencies [8], [9] and for the analysis of drug-drug and drug-gene interactions [10]–[12].

We introduce the eruption plot, an intuitive visualization of strength and significance of interaction effects on a genome-wide scale for the purpose of unraveling non-additive biological mechanisms. For the illustration and testing of our methodology we chose a model data set based on a three-factorial design. In this transcriptomics study the effects of an *in vitro* infection of mouse macrophages from the genetic background C57BL/6 and BALB/c were compared [13]. Two different strains of the intracellular bacterium *Y. enterocolitica* were applied to the macrophage cultures in the presence or absence of the activating cytokine IFN-γ (Table 1 for all used combinations of factors). The three factors under consideration are therefore genetic background of the mice *H* (C57BL/6 or BALB/c), cytokine stimulation *Γ* (application of IFN-γ or no stimulation), and *Y. enterocolitica* infection *I* (control strain WA(pTTS,p60) or infectious strain WA(pYV)). We suggest the eruption plot as a complement to tests like GlobalAncova for the inclusion of significant interactions between covariates. In our application, we demonstrate its relevance for the detection of effect modification and confounding in linear models.

**Table 1:** Experimental setup.

Genetic background	IFN-γ stimulation	Control strain WA(pTTS, pP60)	Virulent strain WA(pYV)	Mock
C57BL/6	No	3	3	3
BALB/c	No	3	3	4
C57BL/6	IFN-γ	3	3	3
BALB/c	IFN-γ	3	3	4

The table summarizes the number of replicates per group. The microarray data comprises genetic background of the mice (C57BL/6 and BALB/c), IFN-γ stimulation, and two *Y. enterocolitica* strains. The *Y. enterocolitica* strain WA(pTTS, p60) is a non-virulent bacterial strain and WA(pYV) is a virulent strain. The non-virulent strain has been engineered as a derivative of WA(pYV). Mock has no infection and serves as a control.

## Materials and Methods

### Eruption Plots

**Figure 2:**
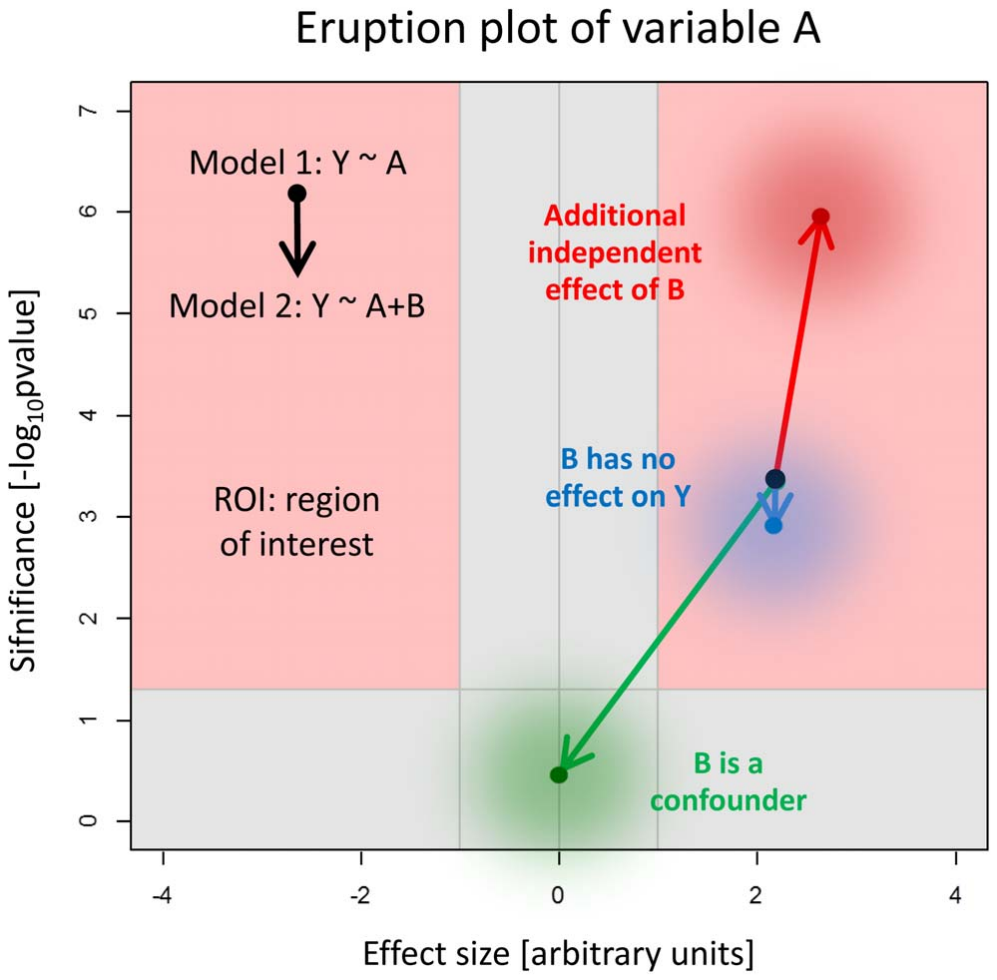
Schematic visualization for the interpretation of the eruption plot. The results of two models can be compared in the eruption plot. The arrows of an eruption plot can have different sizes and directions. This scheme helps to interpret the arrow. Effect size is displayed along the x-axis and the significance on the y-axis. The red area shows the region of interest (ROI).

Volcano plots are commonly used for visualizing the effect size (e.g. expression changes) and significance (p-values of a related statistical test) of a certain variable *A*, if these were estimated for a large number of items (e.g. genes). Each item is represented by a dot showing effect size on the x-axis (e.g., expression fold on a log_2_ scale) and its significance on the y-axis (p-value on a log_10_ scale) [14]. The eruption plot is essentially an overlay of two volcano plots of the variable *A* that were generated from identical data, but using two different models (Figure 2). Every item is represented by an arrow, which connects the dot representing this item in the volcano plot of Model 1 with the corresponding dot in the volcano plot of Model 2. Let us consider how eruption plots can be used for the detection of (ir)relevant covariates, confounding, effect modification (interaction), and for model selection.

Let Model 1 be a linear regression model of the dependent (continuous) variable *Y* versus the covariate *A*, for short *Y∼A*. A variable *B* that has additional effects independent of *A* increases the explanatory power of the extended Model 2, *Y∼A+B* in comparison to Model 1, i.e., it substantially reduces the unexplained variance (the “noise”). Thus, the significance of a potential effect in *A* will be increased, while the effect size estimate of *A* will remain virtually unaffected. The eruption plot of variable *A* will therefore show a long arrow pointing approximately straight upward (Figure 2, Figure S1A). If on the other hand *B* has no additional effect, it will merely, by chance, diminish the effect size estimate of *A*, and thereby also its significance. In this case, the direction of the arrow in the eruption plot of *A* points slightly downward and slightly towards the y-axis (Figure 2, Figure S1 B).

Confounding describes the spurious association between the dependent and an independent variable [15] which is caused by an association of a hidden variable (the confounder) with both the dependent and the independent variable. Additionally, the confounder must not lie on a causal path from the independent to the dependent variable. An example is given in Figure S2A, where *Y* is independent of *A*, however both *Y* and *A* are positively correlated to a confounding variable *B* (see File S1). Here, including *B* in Model 2, *Y∼A+B*, will remove all effects that were spuriously attributed to A in Model 1. Hence, the eruption plot of *A* will show an arrow whose head is located close to the origin.

Effect modification (also called interaction) occurs if the effects of the discrete (group) variables *A* and *B* are not additive, i.e., the *B*-group-specific estimates of *A* differ from one another significantly [16]. The eruption plot can also be used to detect interactions (Figure S2B) by comparing the main effects of *A* and *B* in Model 1, *Y∼A+B* with those in Model 2 containing an interaction term, *Y∼A+B+A∶B*. In presence of effect modification the interaction variable *A∶B* increases explanatory power. By what has been said above, the eruption plots of *A* respectively *B* will therefore point straight upwards.

In combination with global tests such as GlobalAncova, eruption plots reveal if an additional variable has explanatory power or not and thus can be used to decide between a larger and a smaller model. A variable without additional explanatory power is omitted from the model, giving preference to the sparser model (Occam's razor [17]). The iterative removal (inclusion) of a variable then leads to a backward (forward) model selection procedure.

### Global test

GlobalAncova offers a general methodology to study how the expression structure within a group of genes is influenced by design aspects of the study (experiment). Gene-wise linear models are used to formalize the relationship of gene expression with phenotypic or genomic covariates. An ANOVA-based sum of squares summarizes the individual gene-wise linear models to a group statement. This provides the name: GlobalAncova. A permutation test and an asymptotic distribution of the test statistics under the null hypothesis are available to calculate *P*-values. GlobalAncova considers a broad range of designs by exploiting the full scope of linear model theory. We applied GlobalAncova [5] to compare two linear regression models (using 1000 permutations for p-value calculation). The results of the global test were compared with the results of the eruption plot.

### Experimental setup

The published transcriptomics data were generated from bone marrow-derived mouse macrophages [13]. It comprises three different experimental factors (Table S1): *H*, the genetic background of mouse macrophages which is either BALB/c or C57BL/6; *Γ*, an indicator of the presence or absence of IFN-γ cytokine stimulation; *I*, the bacterial strain used for *Y. enterocolitica* infection (virulent strain WA(pYV), control strain WA(pTTS, p60, or mock, no infection). The non-virulent strain was engineered in [18] as a derivative of WA(pYV). Table 1 comprises the number of replicates and shows the combinations of the experimental factors in each microarray experiment. The microarray data is accessible under GEO accession no. GSE 9273 (Table S1 for the single experiments).

### Cluster, pathway, and transcription factor binding site analysis

For every gene the estimated effect of the interaction covariate *H∶Γ* was taken to select for either an alleviating or aggravating effect. If the effect of *H∶Γ* had the same sign as the effect of *H* and of *Γ*, this interaction was interpreted aggravating; if the effect of *H∶Γ* had the opposite sign of the common sign of *H* and of *Γ*, this interaction was considered alleviating. The estimated effects of the three covariates *H* and *Γ* and their interaction *H∶Γ* were subjected to hierarchical clustering and displayed in a heatmap. Only genes showing a significant global effect (F-test <0.05 after FDR correction and at least one of the covariates having an effect estimate of +/−1.5) were subjected to the hierarchical cluster analysis. The dendrogram was taken to order the p-values in a heatmap. Each of the three covariates (*H*, *Γ*, *H*∶*Γ*) can either be positive or negative, resulting in eight different clusters. These gene clusters provided the template for further gene ontology analysis. The clusters were subjected to the DAVID bioinformatics suite [19]. The genes for the transcription factor bindings site (TFBS) analysis were further filtered for a minimum absolute effect size of 0.5 for the covariate *H∶Γ*. For the TFBS analysis the promoter sequence (−500 to +100 bp relative to the transcriptional start site according to the ENSEMBL database) was assembled using the Regulatory Sequence Analysis Tool [20]. For each cluster over-represented TFBSs were predicted using the Transcription Factor Matrix Explorer [21]. The putative TFBSs were taken from the TRANSFAC database [22]. All settings and thresholds were used as in [23].

## Results and Discussion

Using eruption plots we assessed the benefit of comparing linear models by eliminating covariates. In our case study we focused on the genome-wide transcriptional response of different mouse breeds to infection with *Yersinia* in the presence or absence of IFN-γ stimulation (Methods).

### Interaction models improve understanding of transcriptional effects

C57BL/6 mice are able to control and eliminate infection with *Yersinia*. In contrast, BALB/c mice without IFN-γ stimulation succumb to the infection. Resistance against *Yersinia* was shown to correlate with strong induction of IFN-γ early during infection in BALB/c mice [24], [25]. Hence, with respect to survival there is an interaction between IFN-γ and the genetic background. The transcriptional response underlying this interaction and difference in IFN-γ production remains unclear. We therefore investigated interactions on a molecular (the transcriptional) level with a linear model (Model 1, Table 2). We verified that *H∶Γ* contributed significantly to explaining our data, as can be read off the volcano plot of the *H∶Γ* effects [26] (Figure S3).

**Table 2:** Linear regression models.

Model name	Linear regression model
Model 1	*Y∼H+Γ+H∶Γ+I+H∶I+Γ∶I+* ***H*** *∶* ***I*** *∶* ***Γ***
Model 2	*Y∼H+Γ+H∶Γ+I+* ***H*** *∶* ***I*** *+Γ∶* ***I***
Model 3	*Y∼H+Γ+H∶Γ+* ***I***
Model 4	*Y∼H+Γ+H∶Γ*

The table shows the linear regression models, which are tested on the *van Erp* dataset. The linear regression models hold the variables genetic background *Η*, IFN-γ stimulation *Γ*, and the bacterial strain *I*. The dependent variable *Y* is given by gene expression matrix. The fat letters symbolize the additional variables in the model.

### Selecting between nested models using eruption plots

We successively reduced Model 1 by third-order interaction *H∶I∶Γ* (Model 2), then by the interactions of *I* with *H* and *Γ* (Model 2), and finally by the infection variable *I* (Model 4). The hierarchical order of the models allowed us to apply a backward model selection strategy. We started with Model 1 and moved down the hierarchy, successively eliminating covariates as long as we observed an improvement according to our selection criterion. Our main objective was the effect of the inclusion/exclusion of covariates on the estimates of the interactions *H∶Γ*. We used the eruption plot to compare the interaction covariate *H∶Γ* between two models.

**Figure 3:**
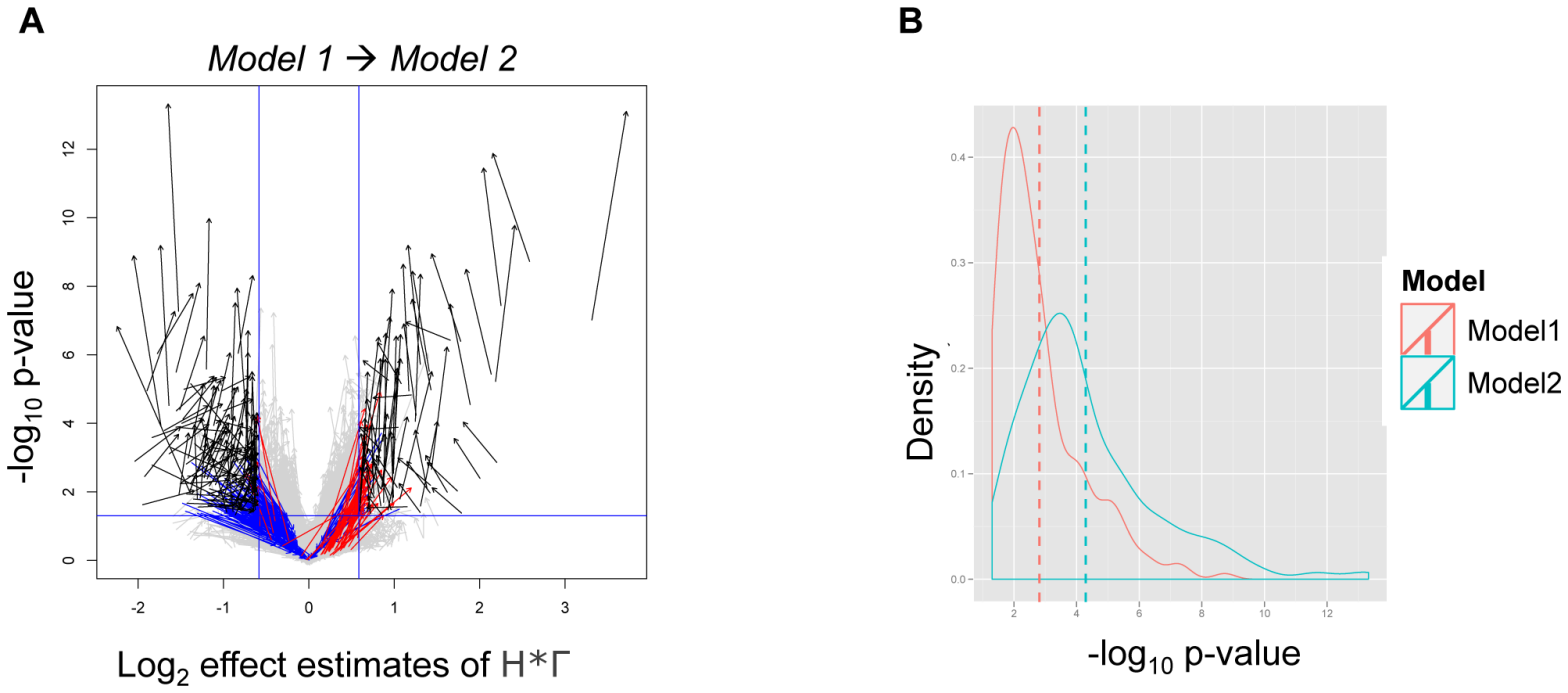
Eruption plot. A: Effect size is displayed along the x-axis at log_2_ scale and the y-axis shows the negative log_10_ p-value. The vertical blue lines indicate 1.5 fold up and down-regulation and the horizontal blue line indicates a significance of 0.05 after Bonferroni adjustment. They bound the regions of biological interest (ROI), which are characterized by a sufficiently high effect, and a sufficiently low p-value. I.e., biologically interesting effects are found in the top left and the top right segment of the plot. Each gene is represented by an arrow comparing the effect size and significance estimate of a covariate (the interaction covariate *H∶Γ* in this case) between Model 1 (arrow tail) to Model 2 (arrow head). The details of Models 1 and 2 are given in Table 2. Black and grey arrows represent genes completely contained within ROI and excluded completely from ROI, respectively. Red and blue arrows represent genes that are located within ROI solely in Model 1 and Model 2, respectively. B: Density plot of the p-values of Model 1 (red) and Model 2 (green). The dashed lines indicate the median of each density.

We tested if the third order term *H∶I∶Γ* disrupted the effect of the interaction covariate. Therefore, we went one step down in the model hierarchy and compared by the eruption plot the results of Model 1 and Model 2 (Figure 3A). The arrows start at Model 1 and end in Model 2. The direction of the arrows showed increased statistical power and a change of effect sizes of Model 2. We also quantified the p-values of the interaction covariate *H∶Γ* of both models by a density plot (Figure 3B). This plot showed higher significance of Model 2. This is also supported by the results of GlobalAncova (p-value = 0.27).

### Assessing explanatory power

We next tested the explanatory power of the second order interaction terms on the interaction covariate. We went one step down in the model hierarchy (Table 2) to set up Model 3. This model included the four main covariates and the interaction covariate *H∶Γ*. We tested if we gain or lose explanatory power by eliminating the second order terms by comparing the results of Model 2 to the results of Model 3 (Figure S5A). We observed no significant difference in statistical power and effect size between both models. The density plot supports these results. Accordingly, the results of GlobalAncova showed no high significance of the second order covariates (p-value = 0.02). Hence, the inclusion of additional second order terms does not improve the model fit. Due to general model selection criteria (Occam's razor) preferring the sparser model we chose Model 3 for upstream analysis.

Our data set also included samples subjected to infection with different *Y. enterocolitica* strains (Table 1). Even though we were mainly interested in differential co-expression of the genetic background and IFN-γ, we included the data of all microarrays into our analysis. We tested the additional explanatory power of the covariate *I*. Model 4 contains only two main covariates *H* and *Γ* and their interaction covariate. The arrows in the eruption plot point from Model 3 to the Model 4 (Figure S5B). The direction of the arrows indicated a small change in p-values. The effect sizes did not change between both models. The density plot stressed the difference between Model 3 and Model 4 and showed an increased statistical power of Model 3. The global test shows the same result, the effects of the two main covariates are significant (p-value = 0,00). Therefore, we gained statistical power by including *I* as a covariate. Consequently, Model 3 was chosen for further analysis and biological interpretation.

### Interaction effects in another double-factorial dataset

**Figure 4:**
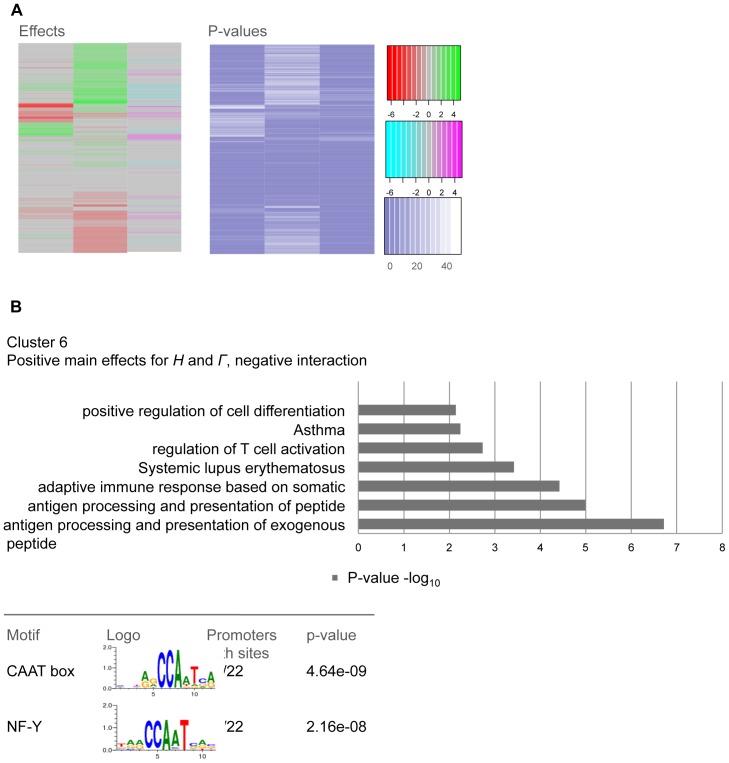
Cluster and pathway analysis. A: the effect estimates of Model 3 were subjected to a hierarchical cluster analysis. Genes are displayed in the rows, which showed a significant global effect (F-test p-value <0.05 after FDR correction and at least one of the covariates having +/−1.5 fold change). The three columns are the covariates *Η*, *Γ*, and *Η∶Γ*. The column *strain* shows differences between C57BL/6 and BALB/c, up-regulation shown in red and down-regulation shown in green. The column *Γ* shows in red up-regulation in BALB/c and in green down-regulation upon IFN-γ stimulation. The third column helps to distinguish alleviating and aggravating effects. Aggravating effects are represented in pink and alleviating effects in turquoise. P-values are plotted separately in a heatmap. The order of the genes is given by the effect estimate clustering. P-values are given in −log_10_ scale and start from 0 displayed in colors ranging from blue to white. B: The results of a pathway enrichment analysis of cluster 6 as a bar plot. The direction of regulation of the genes of cluster 6 is indicated by the color bar. Gene Ontology ‘Biological Process’ terms and KEGG pathway categories (p<0.01) are sorted from bottom (most significant) to top. To reduce redundancy, similar terms are represented by the most significant and specific term. For complete list of functional annotations see Table S2. The right side shows the results of a TFBS analysis of this gene cluster. The two most significantly represented TFBS are given by the name of the transcription factor, the motif, and the p-value.

In order to show that interaction effects are common in microarray experiments with a multi-factorial design, we analyzed an additional multi-factorial data set (GEO accession no. GSE22094). The gene expression data (*Y*) comprises wild type, Fancc-deficient, Fancg-deficient (Fanc and Fancg are nuclear core complex proteins), and double deficient (Fancc/Fancg) mouse macrophages hereafter described by two binary covariates *ΖΓ* (Fancc-deficient) and *ΖH* (Fancg-deficient). We applied two linear regression models: *Y∼ΖΓ+ΖH+ΖΓ∶ΖH* and *Υ∼ΖΓ+ΖH*. We compared the results of the two models by means of an eruption plot of *ΖΓ* (Figure S4A) and *ΖH* (Figure S4B). The arrows in Figure 4A show changes in p-value and in effect size in favor of the first model. This observation is supported by a GlobalAncova test (p-value = 0.01). The eruption plot and the p-value density plot in Figure 4B are partially inconsistent with this. They show changes in favor of the second model. However, the number of genes supporting the second model is substantially smaller. Thus, the interaction covariate reveals effect modification.

### Interaction analysis depicts functional gene clusters

After selecting the appropriate linear regression model for our data, we aimed to analyze the genes showing interaction effects between *H* and *Γ*. We subjected the main effects *H* and *Γ* along with their interaction effect of Model 3 to a hierarchical cluster analysis (Methods) and displayed the result in a heatmap (Figure 4). The first column showed the main effects of the covariate *H* that are conceived as the difference between the predicted gene expression within the macrophages from BALB/c and C57BL/6 background in absence of IFN-γ stimulation. Likewise, the second column *Γ* presents the differences of IFN-γ stimulation in genes within cells of BALB/c background. The genes were selected by the threshold F-test p-value <0.05 after FDR correction and at least one of the covariates having effect estimate of +/−1.5. In the third column aggravating and alleviating interaction effects are indicated in pink and turquoise. The p-values of the genes are plotted accordingly to the sequence of the effect estimate heatmap. The values range from blue to white. The higher the values of the effect estimates are the more significant are the p-values.

Each of the three covariates of this analysis had either positive or negative values and thus a gene could fall into one of eight different clusters. We looked at functional characteristics of the eight clusters by an enrichment analysis of Gene Ontology (GO) terms (biological process) and KEGG pathways (Figure S6). The most significant (p< = 0.01) categories are displayed as bars, sorted from the bottom (most significant) to the top. Similar terms are represented by the most significant and specific term. Table S2 shows a complete list of functional categories. The majority of clusters showed expectedly *immune response* as the most enriched term. Interestingly, cluster 3 shows *cell migration* and *motility of cells*. *Response to wounding* and *defense response* was referred to cluster 7. Cluster 6 showed *antigen processing and presentation via MHC class II* (Figure 4B). We chose this cluster, which includes the antigen-presenting MHC class II genes Aa, Ab, and Eb, for further assessment by *in silico* promoter analysis by TRANSFAC (Figure 4C). This analysis revealed a number of genes, which shared NFY binding sites and the CAAT box. Other genes of cluster 6 such as TRIM30d, IIGP1, and CXCL9 are involved in the IFN-γ -induced immune response. Also the apoptotic regulator Cflar, known also as Flip, is located in this cluster. In our TFBS analysis of these genes we extracted pairs of sites that were found to be enriched in their promoter sequences. We identified NFY binding sites as well as the CAAT boxes as co-localized TFBS in close proximity to the transcriptional start of the genes in this cluster. It seems therefore likely that the transcription factors that bind to this site are responsible for the similar behavior in our expression analysis and thus their placement in cluster 6. This notion is further supported by the fact that an NFY binding site is part of the MHC class II enhanceosome. It seems possible that some of these genes may also constitute C2TA targets and are therefore co-regulated with MHC class II.

### Interaction analysis discovers biologically relevant features

To show the biological value of the interaction analysis we chose the gene H2-Ea, which is an MHC class II gene that is under control of IFN-γ through the C2TA transactivator protein. H2-Ea is an active gene in BALB/c; its product forming the surface-expressed peptide-presenting H2-E heterodimer. It is a pseudogene (H2-Ea-ps) in C57BL/6 due to a large genomic deletion that includes the core promoter and the transcription start site. Thus, in mRNA of macrophages of C57BL/6 background generally no transcript of H2-Ea-ps is found. Therefore, this gene can be seen as a genetic example for cluster 8, an alleviating interaction: a) transcript levels in C57BL/6 should be vastly reduced in comparison to BALB/c; b) IFN-γ should not have any influence on expression of the pseudogene in C57BL/6 macrophages. A detailed analysis of the expression pattern of this gene is shown by a scatter plot in Figure S7. In our expression analysis we observed that gene expression of H2-Ea in BALB/c is up-regulated upon IFN-γ stimulation. Further, we found low gene expression of H2-Ea in C57BL/6, with and without IFN-γ stimulation. Expectedly, inclusion of the bacterial covariates *I_1_*, and *I_2_* does not deliver additional explanatory power (Figure S7).

While Ea is a special case due to its nature as a pseudogene in one of the analyzed genetic background of the mice, the MHC class II genes that are functional (Eb, Aa, and Ab), as discussed above, were allocated into clusters 6. The MHC class II genes were found to be up-regulated in C57BL/6 and IFN-γ. Their interaction effect presented as alleviating since the expression in IFN-γ treated macrophages of C57BL/6 mice was lower than expected from the effect sum of the two single covariates. It has been known for a long time that the regulation of MHC II expression is almost exclusively dependent on the binding of the transcriptional transactivator C2TA to a constitutive, yet inactive, enhanceosome complex including RFX-AP, -ANK, -5, CREB, and NF-Y [27]. Therefore it would appear logical to find C2TA in the same clusters as the MHC class II genes (Table S2, cluster 6). Yet, unexpectedly we find C2TA in clusters 2 and 4, which show both an increase of C2TA mRNA by IFN-γ and an aggravating interaction effect. A closer analysis of the data reveals however, that this outcome is result of a very small expression change between the genetic background of the mice. Since both probe sets for C2TA recognize the same (and main) exon of C2TA this result can only be explained by noise. Thus, we can assume C2TA expression to be basically unchanged between the strains, with a strong effect seen by IFN-γ. This strong effect is more pronounced in C57BL/6 mice than in BALB/c mice. The effect of IFN-γ-mediated C2TA up-regulation is reflected in the expression increase of the classical functional MHC class II genes Eb, Aa, and Ab as well. While this is expected by the biology of expression control of MHC class II genes, it is interesting to note that the interaction effect was calculated as alleviating. It can be postulated that the IFN-γ and thus in turn C2TA-mediated increase in the transcription of the MCH class II genes runs into the ceiling of possible transcription at that locus. Thus, the expression difference found between the strains in steady-state cannot translate into an effect in the presence of IFN-γ.

## Conclusions

In this study higher order linear regression models were applied to microarray expression data in order to identify interactions between multiple treatments and their effects on the transcriptional response. The aim of our study was to establish the eruption plot as a valuable auxiliary tool for model selection in a hierarchy of models. While GlobalAncova can be used to assess a difference in fit between two models, giving a p-value to test the null hypothesis of equal fit, GlobalAncova does not provide any insight how the different covariates contribute to the fit. The eruption plot was developed to visually select the best model for the given, high-dimensional data. The prevailing directions of the arrows an eruption plot can uncover effect modification, confounding, as well as an improvement of explanatory power of a covariate. Applying this methodology to microarray data from different mouse breeds that were infected with different agents and received INF-γ stimulation or not, we show that second order effects are present in the data set. We conclude that higher order interaction effects should always be considered when linear regression models are applied to multi-factorial microarray data. The biologically interesting interaction effects of mouse breed and INF-γ stimulation were qualitatively interpreted and classified into neutral, alleviating, or aggravating effects. A clustering of genes based on their effect sizes resulted in eight gene clusters which were subjected to a pathway and TFBS analysis. We found one gene cluster built up of putative C2TA targets which are co-regulated with MHC class II genes, indicating the biological significance of our approach.

## Supporting Information

Figure S1 **Model selection by the eruption plot.** A: The response *Υ* is the sum of the covariates *A* and *B* and a noise term. The eruption plot compares the effect estimates for covariate *A* in a linear model containing only covariate *A* (arrow shaft) with that of the correct linear model (arrow head). B: The response *Y* is the sum of *A* and a noise term. The eruption plot compares the effect estimates for covariate *A* in the correct model (arrow shaft) with a linear model including *A* and *B* (arrow head).
(TIF)

Figure S2 **Examples for confounding and effect modification.** A: the upper plot shows a scatter plot of noisily increasing data. The arrow of the lower plot shows the comparison of *Y∼A+B* to model *Y∼A*. B: the upper plot shows a scatter plot of noisy data. The arrow of the lower plot shows the comparison of *Y∼A+B* to model *Y∼A+B*+*A∶B*.
(TIF)

Figure S3 **Volcano plot of Model 4.** Linear regression model includes estimation of the effects as given in Model 4 (Table 2). The volcano plot displays the effects of interaction covariate *Η∶Γ*. The log_2_ fold change is displayed on the x-axis and the negative log_10_ p-value is displayed on the y-axis.
(TIF)

Figure S4 **Eruption plot of a double-factorial dataset.** The data *Υ* comprises two single gene-deletions Fancc *ΖΓ* and Fancg *ΖΗ* one double gene-deletion of Fancc and Fancg. A: the left plot shows an eruption plot, comparing covariate *ΖΓ* of the two models: *Υ∼ΖΓ+ΖΗ+ΖΓ∶ΖΗ* (shaft) *Υ∼ΖΓ+ΖΗ* (head). The right plot shows the corresponding histogram of the p-values from covariate *ΖΓ* of both models. B: the left plot shows the eruption plot of the same models but comparing covariate *ΖΗ*. On the right is the corresponding histogram of the p-values from covariate *ΖΗ* of both models.
(TIF)

Figure S5 **Eruption plots.** Effect size is displayed along the x-axis at log_2_ scale and the y-axis shows the negative log_10_ p-value. Grey arrows show not significant effects of both models and black arrows significant effects of both models (BH corrected p-values <0.05 and fold change >+/−0.5). The blue lines starting from the x-axis are at +/−0.5 and the line starting at the y-axis is at −log_10_ (0.05). The model details are given in Table 2. A: Eruption plot from Model 2 to Model 3: the arrows start at the results from Model 2 and end at the results of Model 3. The arrows are short, so there are no big differences between both models. The density plot next to the eruption plot shows the density of the p-values from both models. B: Eruption plot from Model 3 to Model 4: The arrows point from the results of Model 3 to the results of Model 4. The density plot next to the eruption plot shows the density of the p-values from both models.
(TIF)

Figure S6 **Gene ontology and TFBS analysis.** The gene clusters shown in Figure 4 were subjected to a gene ontology and TFBS analysis. Each cluster is build up by genes having effect sizes of the three covariates *Η*, *Γ*, and *Η∶Γ*. The column *strain* shows differences between C57BL/6 and BALB/c, up-regulation shown in red and down-regulation shown in green. The column *Γ* shows in red up-regulation upon IFN-γ, stimulation in BALB/c and in green down-regulation upon *Γ* stimulation. The third column helps to distinguish alleviating and aggravating effects. Pink color reflects aggravating effects and in turquoise alleviating effects. Functional characteristics of the eight clusters are defined by an enrichment analysis of Gene Ontology (GO) terms (biological process) and KEGG pathways. The left side shows a list of the functional categories belonging to Cluster 1–8. The right side shows the results of the TFBS analysis. The two most significantly represented TFBS are given for each gene cluster along with the name of the transcription factor, the motif, and the p-value.
(TIF)

Figure S7 **Scatter plot of gene expression data.** The scatter plot shows the gene expression data from BALB/c mice and C57BL/6 mice of gene H2-Ea-ps. The form of the data points reflects if the probe was treated with an infection *I* and the color indicates if the probe was stimulated by *Γ*.
(TIF)

File S1 **R code to reproduce the eruption plots to simulate confounding.**
(R) Click here for additional data file.

Table S1 **Design matrix.**
(XLSX)

Table S2 **Functional characteristics of cluster 1–8.**
(XLSX)
