# Supplementary figures and images for: Selection of Higher Order Regression Models in the Analysis of Multi-Factorial Transcription Data
Source: PLoS One. 2014 Mar 21;9(3):e91840. doi: 10.1371/journal.pone.0091840 (PMC3962375; doi:10.1371/journal.pone.0091840)

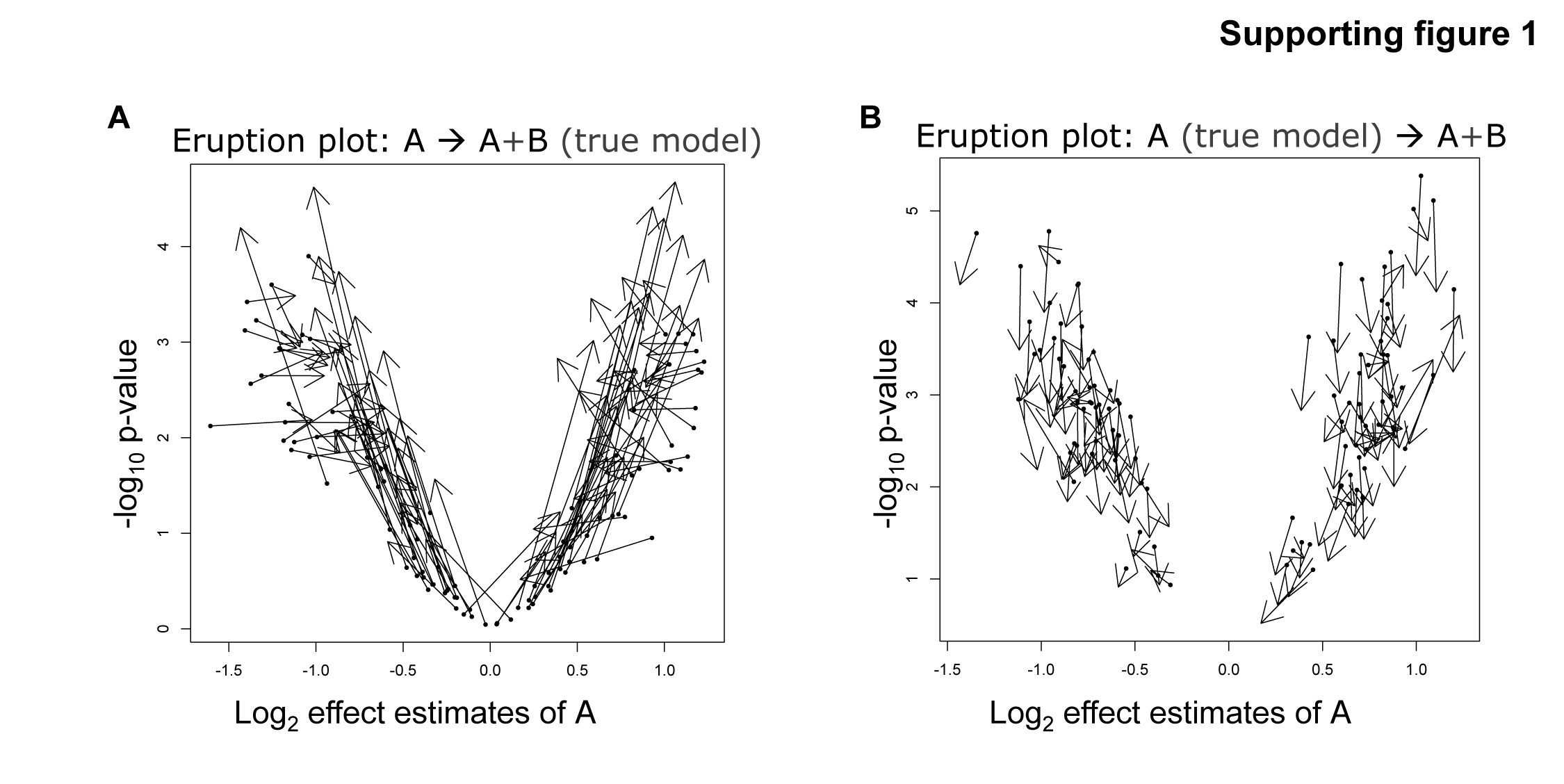

Supplement: Figure S1 — Model selection by the eruption plot. A: The response Υ is the sum of the covariates A and B and a noise term. The eruption plot compares the effect estimates for covariate A in a linear model containing only covariate A (arrow shaft) with that of the correct linear model (arrow head). B: The response Y is the sum of A and a noise term. The eruption plot compares the effect estimates for covariate A in the correct model (arrow shaft) with a linear model including A and B (arrow head). (TIF) [file pone.0091840.s001.tif]

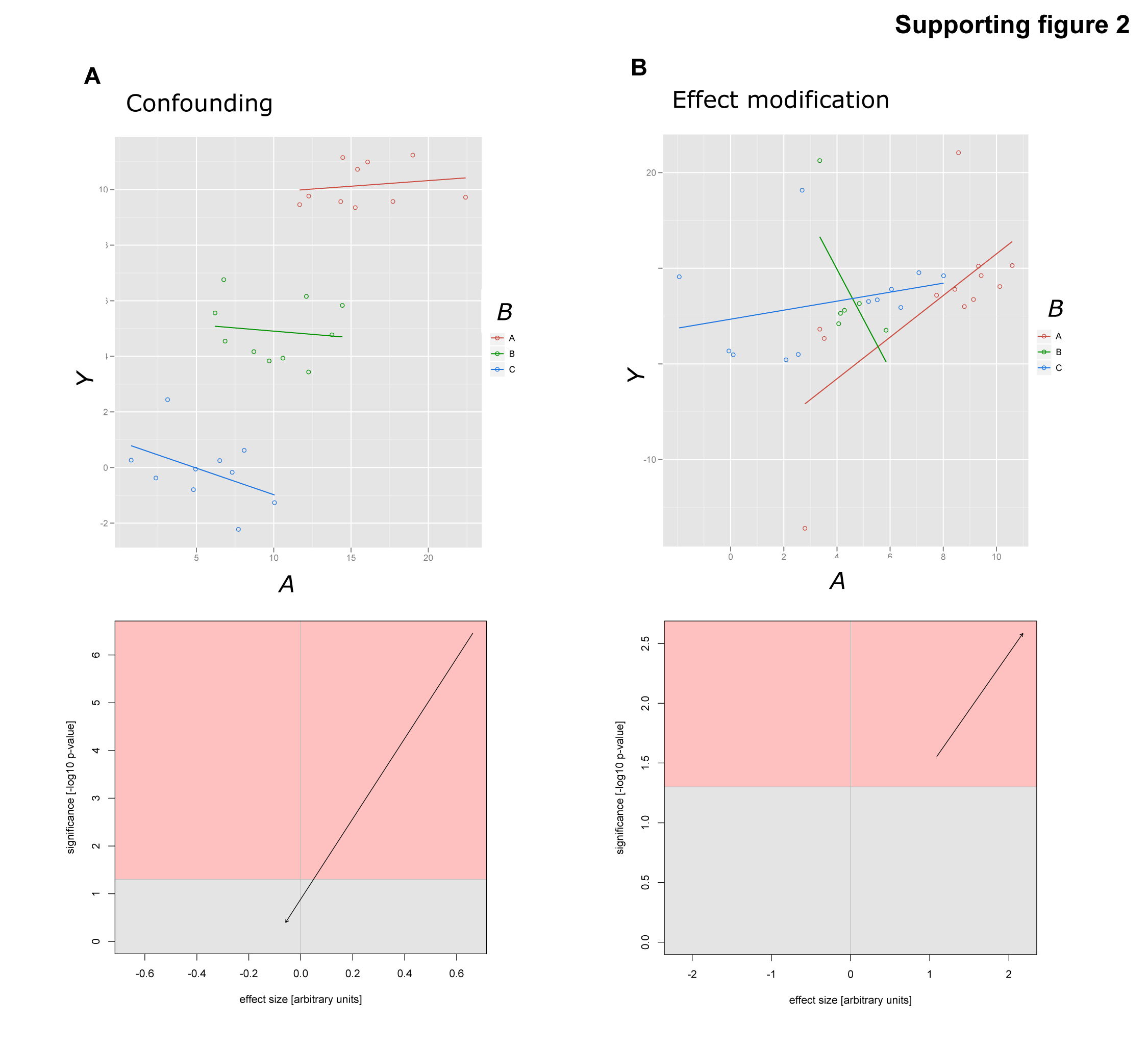

Supplement: Figure S2 — Examples for confounding and effect modification. A: the upper plot shows a scatter plot of noisily increasing data. The arrow of the lower plot shows the comparison of Y∼A+B to model Y∼A. B: the upper plot shows a scatter plot of noisy data. The arrow of the lower plot shows the comparison of Y∼A+B to model Y∼A+B+A∶B. (TIF) [file pone.0091840.s002.tif]

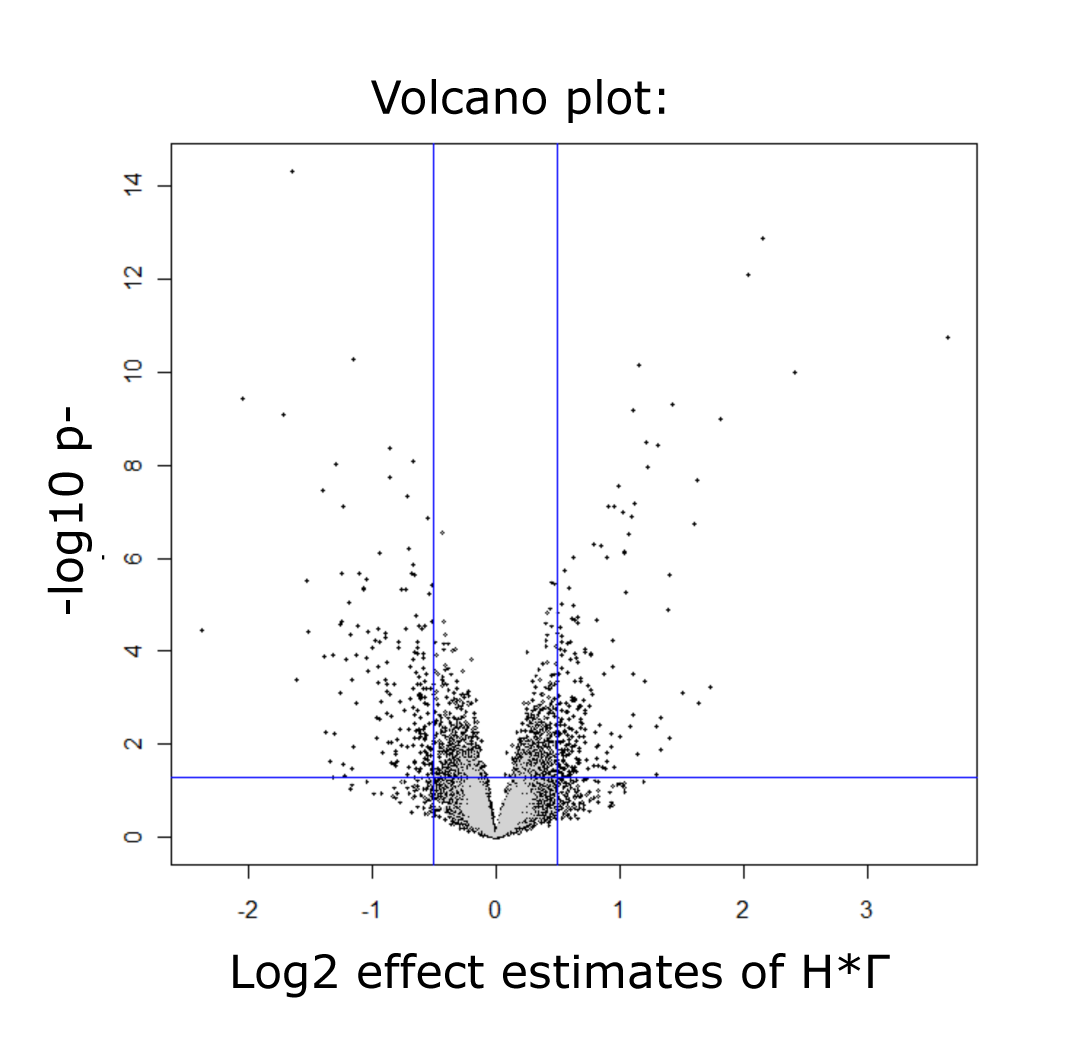

Supplement: Figure S3 — Volcano plot of Model 4. Linear regression model includes estimation of the effects as given in Model 4 (Table 2). The volcano plot displays the effects of interaction covariate Η∶Γ. The log2 fold change is displayed on the x-axis and the negative log10 p-value is displayed on the y-axis. (TIF) [file pone.0091840.s003.tif]

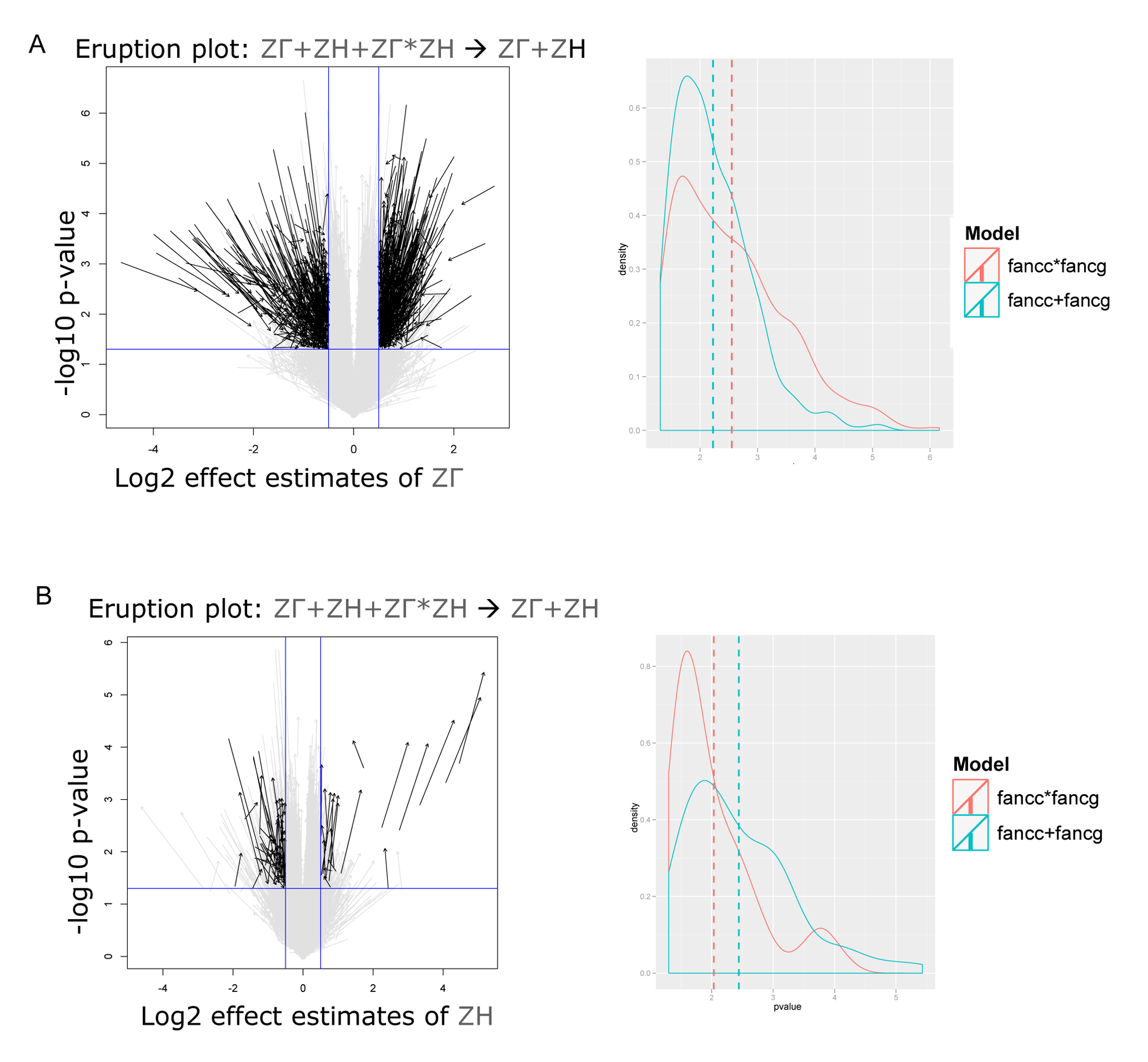

Supplement: Figure S4 — Eruption plot of a double-factorial dataset. The data Υ comprises two single gene-deletions Fancc ΖΓ and Fancg ΖΗ one double gene-deletion of Fancc and Fancg. A: the left plot shows an eruption plot, comparing covariate ΖΓ of the two models: Υ∼ΖΓ+ΖΗ+ΖΓ∶ΖΗ (shaft) Υ∼ΖΓ+ΖΗ (head). The right plot shows the corresponding histogram of the p-values from covariate ΖΓ of both models. B: the left plot shows the eruption plot of the same models but comparing covariate ΖΗ. On the right is the corresponding histogram of the p-values from covariate ΖΗ of both models. (TIF) [file pone.0091840.s004.tif]

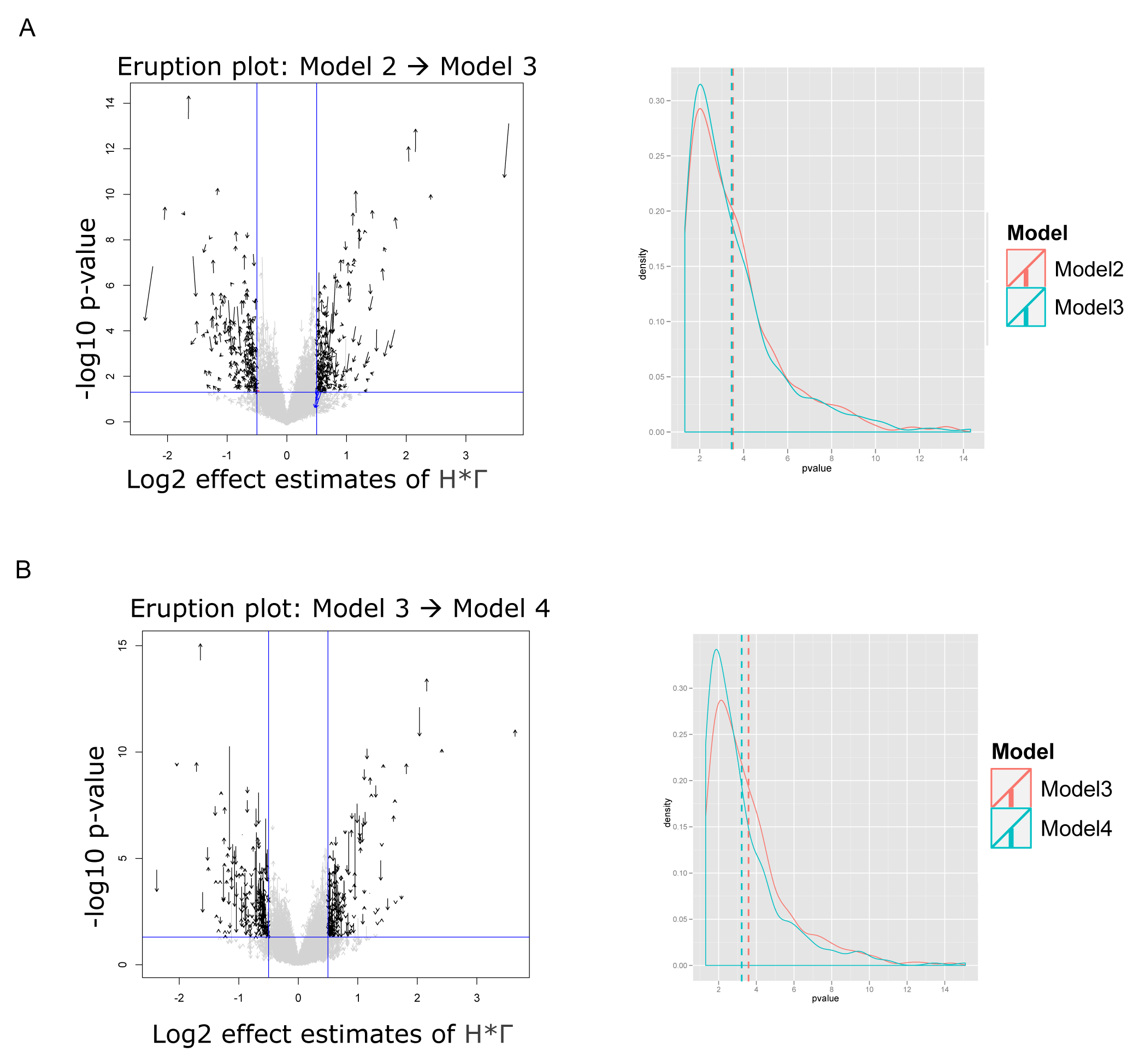

Supplement: Figure S5 — Eruption plots. Effect size is displayed along the x-axis at log2 scale and the y-axis shows the negative log10 p-value. Grey arrows show not significant effects of both models and black arrows significant effects of both models (BH corrected p-values <0.05 and fold change >+/−0.5). The blue lines starting from the x-axis are at +/−0.5 and the line starting at the y-axis is at −log10 (0.05). The model details are given in Table 2. A: Eruption plot from Model 2 to Model 3: the arrows start at the results from Model 2 and end at the results of Model 3. The arrows are short, so there are no big differences between both models. The density plot next to the eruption plot shows the density of the p-values from both models. B: Eruption plot from Model 3 to Model 4: The arrows point from the results of Model 3 to the results of Model 4. The density plot next to the eruption plot shows the density of the p-values from both models. (TIF) [file pone.0091840.s005.tif]

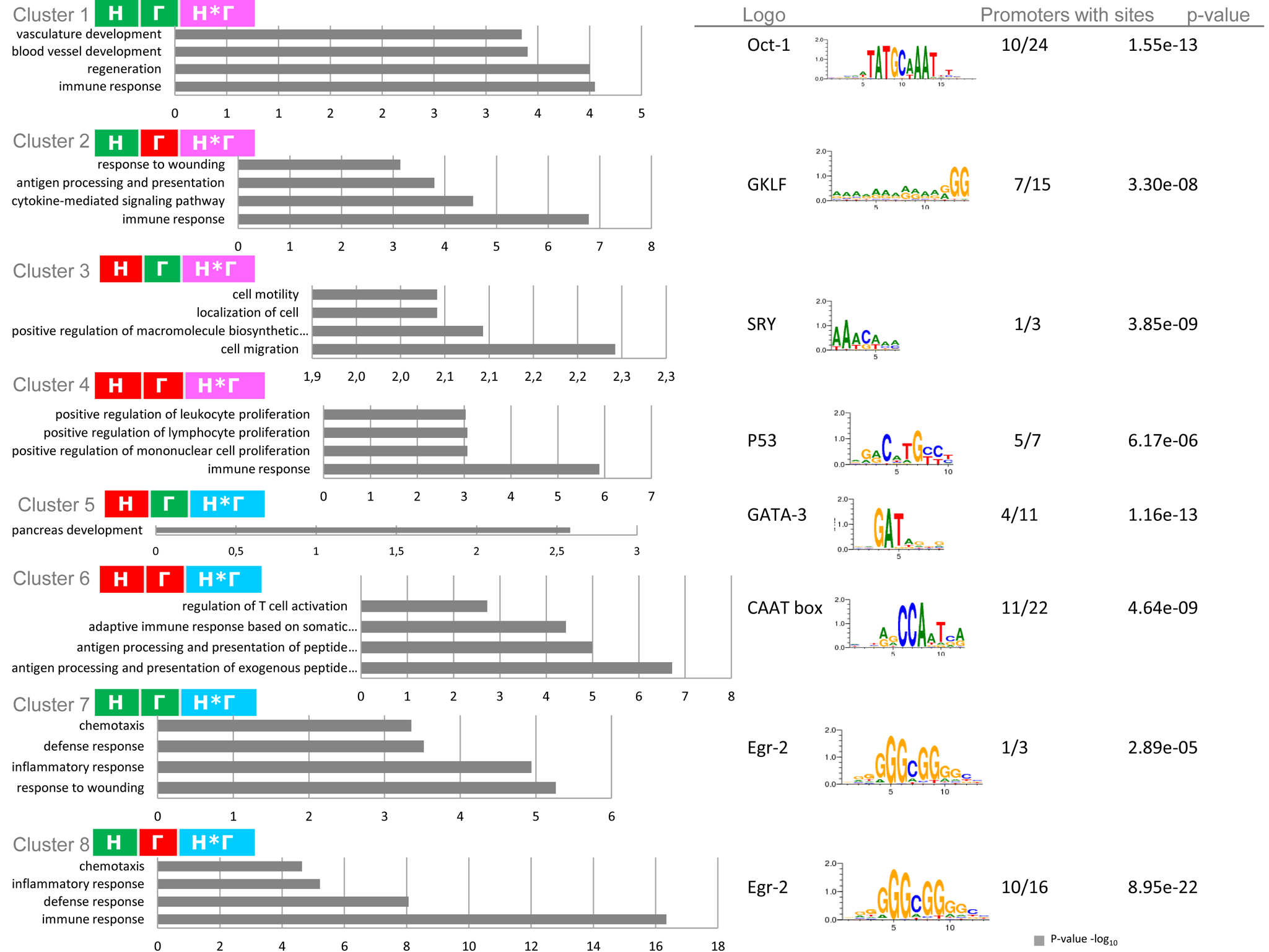

Supplement: Figure S6 — Gene ontology and TFBS analysis. The gene clusters shown in Figure 4 were subjected to a gene ontology and TFBS analysis. Each cluster is build up by genes having effect sizes of the three covariates Η, Γ, and Η∶Γ. The column strain shows differences between C57BL/6 and BALB/c, up-regulation shown in red and down-regulation shown in green. The column Γ shows in red up-regulation upon IFN-γ, stimulation in BALB/c and in green down-regulation upon Γ stimulation. The third column helps to distinguish alleviating and aggravating effects. Pink color reflects aggravating effects and in turquoise alleviating effects. Functional characteristics of the eight clusters are defined by an enrichment analysis of Gene Ontology (GO) terms (biological process) and KEGG pathways. The left side shows a list of the functional categories belonging to Cluster 1–8. The right side shows the results of the TFBS analysis. The two most significantly represented TFBS are given for each gene cluster along with the name of the transcription factor, the motif, and the p-value. (TIF) [file pone.0091840.s006.tif]

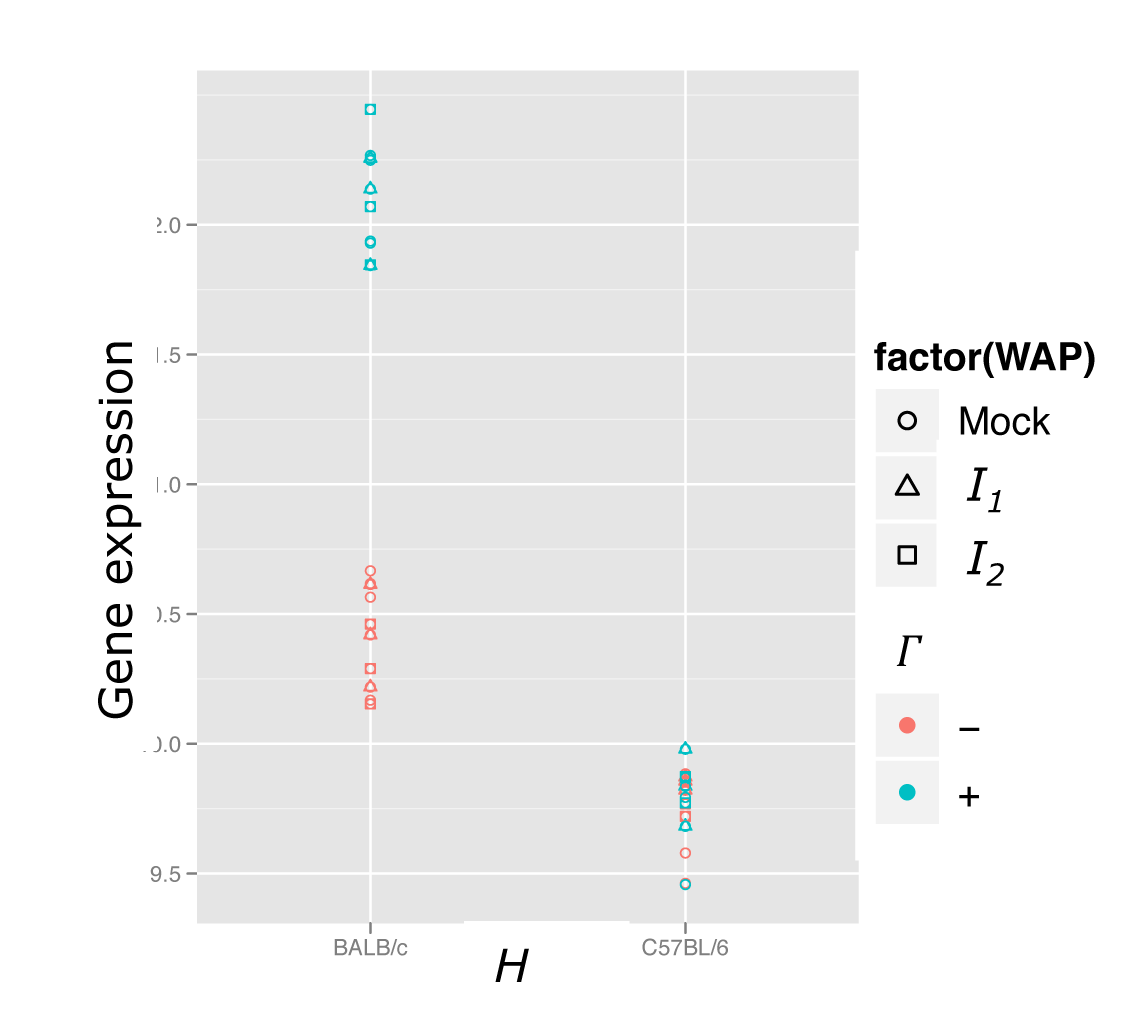

Supplement: Figure S7 — Scatter plot of gene expression data. The scatter plot shows the gene expression data from BALB/c mice and C57BL/6 mice of gene H2-Ea-ps. The form of the data points reflects if the probe was treated with an infection I and the color indicates if the probe was stimulated by Γ. (TIF) [file pone.0091840.s007.tif]
